# Evaluation of the practice of reprocessing ORs in German hospitals from an infection prevention and control perspective

**DOI:** 10.1007/s15010-024-02303-z

**Published:** 2024-06-03

**Authors:** C Grimm, S Scheithauer, T Artelt, A Stieber, J Erlenwein, M Schuster, M Bauer, Reiner M. Waeschle

**Affiliations:** 1 Department of Anesthesiology, University Medical Center Göttingen, Robert-Koch-Str. 40, 37075 Göttingen, Germany; 2 Department of Infection Control and Infectious Diseases, University Medical Center Göttingen, Göttingen, Germany; 3 Clinic for Anesthesiology, Intensive Care Medicine, Emergency Medicine and Pain Therapy, RKH-Kliniken Landkreis Karlsruhe, Fürst-Stirum-Klinik Bruchsal and Rechbergklinik Bretten, Bruchsal, Germany; 4 Clinic for Anesthesiology, Intensive Care Medicine and OR Management, RHÖN- KLINIKUM Campus Bad Neustadt, Bad Neustadt an der Saale, Germany

**Keywords:** Infection prevention and control, Reprocessing, OR, Disinfection, Survey

## Abstract

**Background:**

The aim of this study was to analyze the cleaning and disinfection of operating rooms (ORs) status quo focusing on hygiene plans in German hospitals.

**Methods:**

In 2016, a structured online survey was sent to infection prevention and control (IPC) specialists at the cost calculation hospitals of the Institute for the Hospital Remuneration System (InEK) and all university hospitals in Germany (*n* = 365).

**Results:**

With a response rate of 27.4%, 78% stated that written hygiene plans were available. After cleaning and disinfecting an OR with a “septic” patient, 55% waited until surfaces were dry before reusing in accordance with national recommendations, 27% waited > 30 min. Additionally, 28% of hospitals had ORs only for “septic” patients. In 56% “septic” patients were only operated on at the end of the program. Postoperative monitoring of patients with bacteria with special IPC requirements took place in the post anesthesia care unit (PACU) (29%), operating room (OR) (52%), intensive care unit (ICU) (53%), and in the intermediate care unit (IMC) (19%).

**Discussion and conclusions:**

Despite written hygiene plans in place the partly long duration of OR nonuse time following IPC measures, the consistent continued use of stratification for “septic” patients and the postoperative follow-up care of patients with colonizing/infecting bacteria with special IPC requirements in the OR and high care areas represent relevant potential for improvement.

**Supplementary Information:**

The online version contains supplementary material available at 10.1007/s15010-024-02303-z.

## Introduction

The operating room (OR) is a high-risk and high-cost area [1, 2]. Therefore, measures to improve quality in combination with the optimization of care processes and efficient staff deployment planning are of particular importance [3]. This aspect also applies to infection prevention and control (IPC) measures in the OR to prevent postoperative surgical site infections (SSI). In addition to direct patient-related measures such as the appropriate timing of preoperative antibiotic administration and adequate skin antisepsis, there are measures relating to the immediate patient environment, such as OR reprocessing between surgical procedures, instrument reprocessing and various other measures [4, 5]. Measures relating to OR reprocessing are often based on traditional approaches. Deficits in the planning and implementation of IPC measures in the OR can lead to quality deficits, process delays or increased staff retention times. In addition, hygiene standards in everyday life are still partly characterized by traditions that need to be critically questioned [6].

To address this challenge, a written, agreed upon hygiene plan guided by professional expertise is required. Among other things, this should define clear responsibilities and specifications regarding the deployment of personnel, the measures to be carried out and the processing times for the various cleaning and disinfection procedures. The introduction of a consensual, evidence-based hygiene plan for the OR has been shown to simplify cleaning and disinfection procedures, optimize staff deployment, shorten process times and reduce the potential for interprofessional and interdisciplinary conflict [7–9].

To date, there has been a lack of information regarding the distribution and content of such hygiene plans in ORs in German hospitals and worldwide. For this reason, a nationwide survey was carried out to investigate the distribution of binding hygiene plans, the integration of IPC measures into the process structure of the OR, the handling of multidrug-resistant organisms (MDRO) and personnel resource planning in ORs in German hospitals.

### Background information regarding IPC in German hospitals

Of course, adherence to IPC measures in ORs is not only a matter of medical protocol but also of legal compliance. In Germany, central to this regulatory framework is the German Social Accident Insurance (“Deutsche Gesetzliche Unfallversicherung”, DGUV). The DGUV is the German statutory accident insurance and insures people against the consequences of accidents at work, commuting accidents and occupational illnesses. It is responsible for ensuring that workplaces, including healthcare settings, uphold rigorous safety and health standards. This institution is pivotal in mandating how hospitals implement and maintain IPC measures to protect healthcare workers and patients from occupational risks and healthcare-associated infections. Understanding the DGUV’s role provides a crucial backdrop for discussing IPC practices in German ORs, as it shapes the strategies and responsibilities of healthcare facilities in maintaining high standards of care and safety.

## Materials and methods

### Study population

As part of this survey, IPC specialists, if available, or IPC nurses from the cost calculation hospitals of the Institute for the Hospital Remuneration System (“Institut für das Entgeltsystem im Krankenhaus” (InEK); as of 2014) and from German university hospitals (according to the German Association of University Hospitals, as of 2014) were surveyed (*n* = 365). The relevant contact persons were identified online or via contact with the hospital administration. The online survey was conducted using the online tool Survey Monkey (Momentive Europe UC, Ireland) and was launched on 14 September 2015. Data collection lasted until 1 July 2016. This study used a pragmatic approach by selecting InEK hospitals due to their large, diverse sample and convenient accessibility. These hospitals provide a quasi-representative cross-section of Germany’s healthcare environment, enabling efficient analysis of IPC practices. The focus on InEK hospitals allowed for robust results that potentially apply to the entire German healthcare system.

### Questionnaire

The questionnaire was created by the authors. Independent review and pretesting were carried out by two medical staff members of the Department of Infection Control and Infectious Diseases, University Medical Center Göttingen, and the questionnaire was optimized accordingly. The final version of the questionnaire comprised 42 questions on the following aspects:

1. General data (hospital size, care level, etc.)
2. General aspects of the dissemination and content of hygiene plans.
3. Integration of IPC measures into the process structure of the OR.
4. Special aspects regarding.
  - the handling of patients with explicitly hospital-relevant infections/colonizations (‘septic’ patients definition see below),
  - the handling of colonizing/infecting bacteria/viruses with special IPC requirements (like *Norovirus, Clostridioides difficile, Corynebacterium diphtheriae, Mycobacterium tuberculosis* etc.), and.
  - the handling of MDRO.

The last three subgroups (4 a-c) reflect different clinical situations with great relevance in daily clinical practice. The relevant pathogens clearly overlap in the three subgroups. For example, the pathogens from groups b and c can also be found in group a. The question types consisted of dichotomous questions, questions with 4-point and 6-point ordinal scales and questions with multiple answers. At the end of the questionnaire, there was also the opportunity to provide further suggestions in a free-text field. The time required to complete the questionnaire was approximately 25 min. The responsible ethics committee of the University Medical Center Göttingen was informed (DOK_202_2015). Formal authorization was not required due to the anonymous data collection. Informed consent of the participants was assumed through their voluntary participation after they received detailed information about the survey.

The responding hospitals were categorized into different care levels: primary and standard care hospitals, specialized care hospitals, maximum care hospitals, university hospitals and special care hospitals [10, 11]. Primary and standard care hospitals are those with internal medicine and/or general surgery and at least one other specialist department, usually gynecology and obstetrics, ear, nose and throat medicine, ophthalmology or orthopedics. Specialized care hospitals cover an even broader spectrum of departments, including specialist departments, e.g., pediatrics and neurology. Maximum care hospitals are characterized by offering all specialist areas, and university hospitals additionally specialize in rare diseases and complicated courses of illness within specialist areas. Special care hospitals treat specific illnesses. They can have corresponding specialist or specialty competencies with existing departments [10].

### Definition of “septic” patients

“Septic” interventions or interventions on “septic” patients were defined as surgical interventions on patients who were explicitly colonized and/or infected with hospital-relevant bacteria, regardless of the location of the colonization or infection.

### Survey and mailing list

Initially, the above mentioned groups were contacted by telephone to introduce this study and ascertain their interest in participating. Afterward, the survey was sent by email.

It was possible to contact the authors for questions by mobile phone or e-mail. The survey was not advertised and contained introductory text on the background of the survey (see the supplemental material).

Four automated reminders were sent via Survey Monkey to respondents who had not yet completed the survey. By the end of the data collection, 100 questionnaires had been answered. There was no financial or nonmonetary compensation for participation. The results of the survey were made available to the participants upon request. The data were analyzed using the online tool Survey Monkey and in Excel 2016 (Microsoft©, USA). The survey was conducted and analyzed in accordance with the parameters of the CHERRIES checklist, insofar as they were applicable to our survey [12].

### Descriptive statistics

Multiple answers were permitted for various questions, and the number of answers given was related to the number of questionnaires answered; this potentially led to a cumulative percentage of more than 100% for this type of question.

The descriptive statistical analysis was carried out using nonparametric methods.

## Results

### General questions

A total of 100 out of the 365 hospitals identified took part in the survey, which corresponded to a response rate of 27.4%.

Of a total of 100 responding hospitals, 41% were primary and standard care hospitals, 19% were specialized care hospitals, 17% were maximum care hospitals, 13% were university hospitals, and 9% were special care hospitals.

A total of 78% of the hospitals stated that written hygiene plans were in place. In 79% of hospitals, the hygiene plans used were identical for all the departments involved.

### Main OR for procedures on “septic” patients

A total of 28% of the hospitals stated that they had one or more ORs exclusively for the treatment of patients with what they considered to be explicitly hospital-relevant bacteria (“septic” OR). 68% of the respondents did not have such ORs in their hospital, while 4% did not provide any information. 70% of the participating hospitals stated that patients with what they considered to be explicitly hospital-relevant infections also underwent surgery outside of special “septic” ORs, although these were provided.

**Fig. 1:**
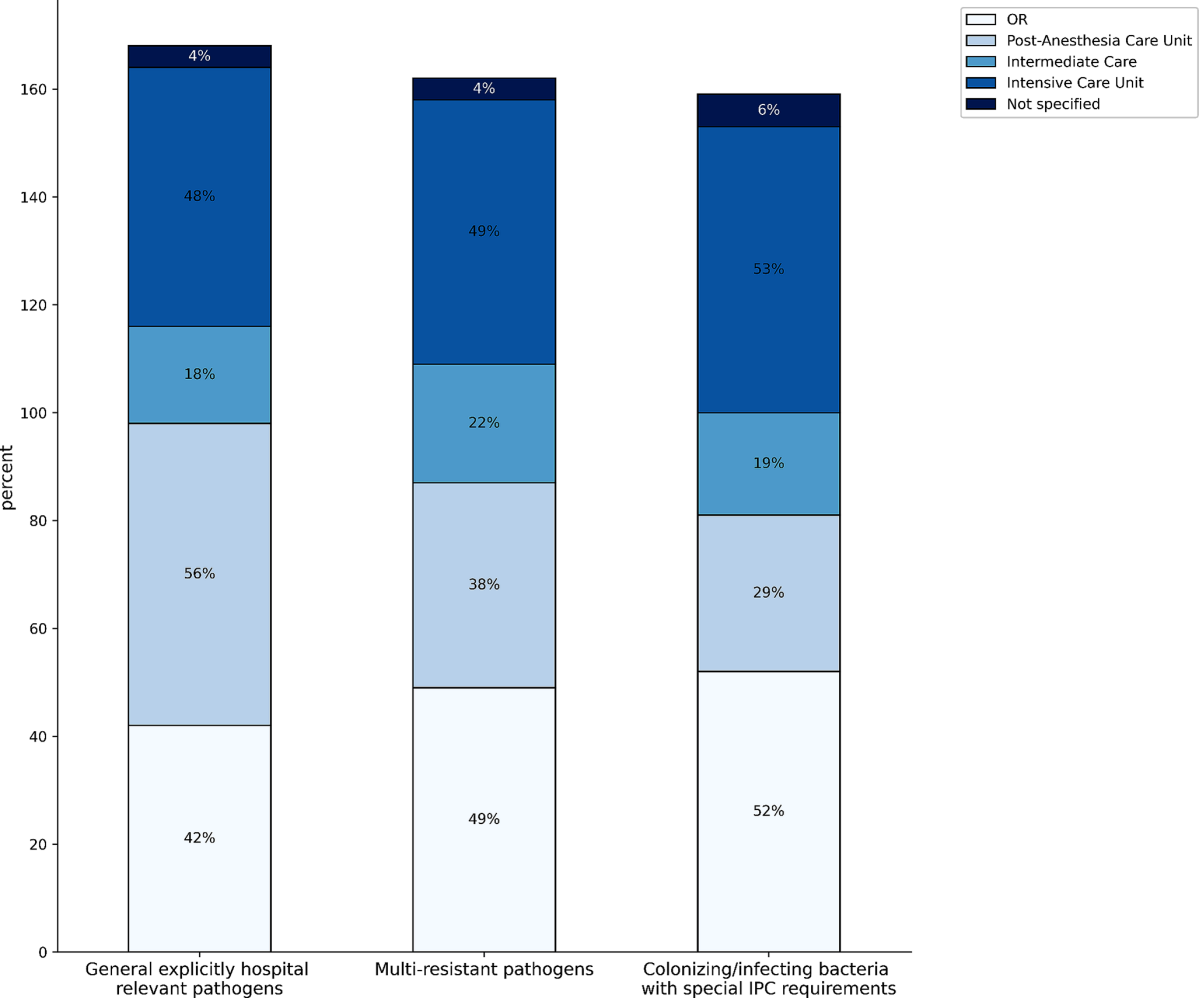
Differentiation of postoperative procedures for “septic” patients with bacteria that are explicitly hospital relevant according to their assessment

The differences in procedures after surgical interventions for “septic” patients are shown in Fig. 1.

### Time of surgery for “septic” patients

**Fig. 2:**
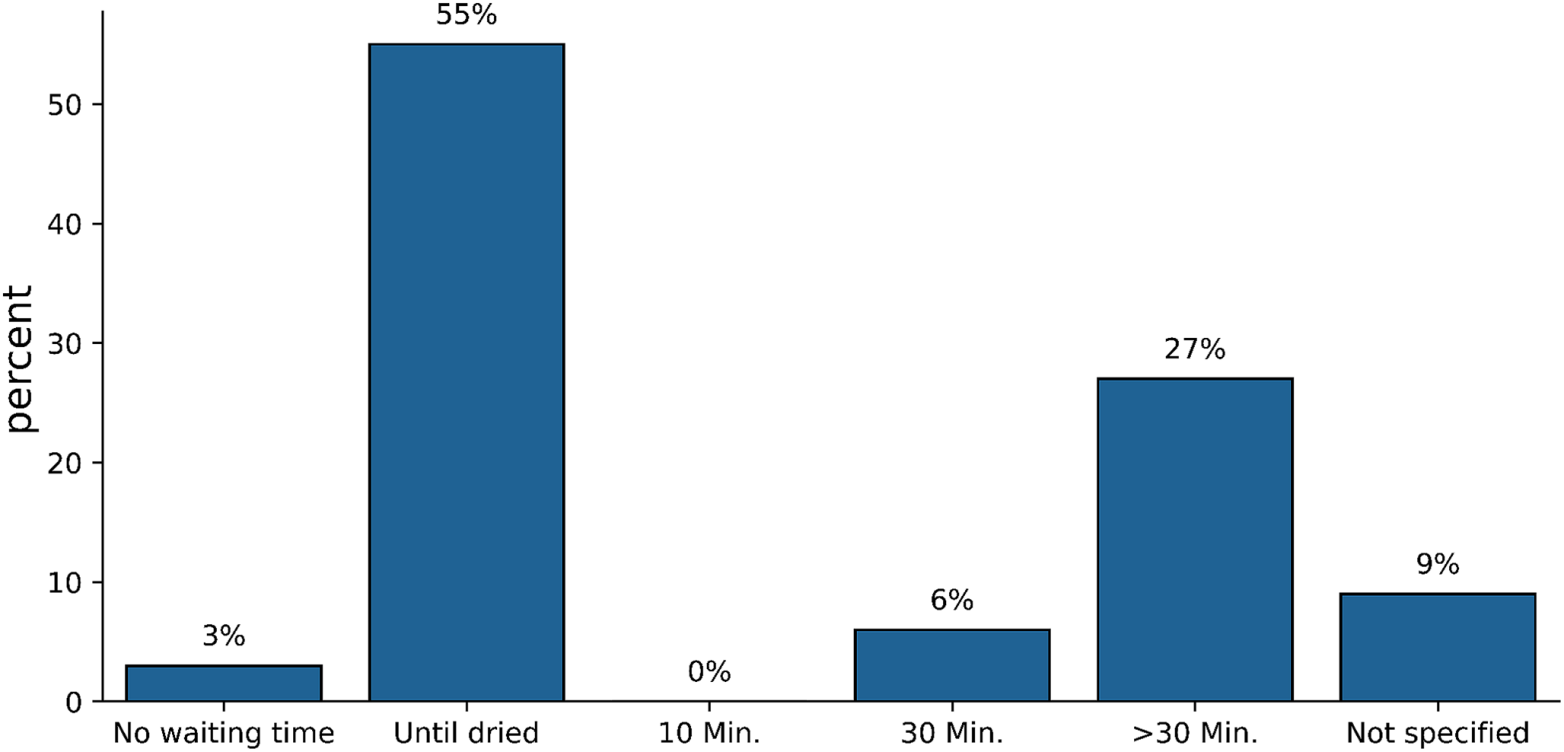
Duration of nonuse specified in the hygiene plan due to exposure time, if applicable, the OR nonuse period after OR preparation as a result of “septic” interventions

56% of the participating hospitals stated that they operated on patients who were infected with what they considered to be explicitly hospital-relevant bacteria only at the end of the daily OR program, regardless of the location of the colonization or infection. A total of 25% of hospitals reported that they treated these patients at any time during the daily OR program. In 10% of hospitals, “septic” patients were only treated without subsequent surgery during the course of the day. 6% did not provide any information. The durations of nonuse of the hygienically prepared ORs for “septic” patients are shown in Fig. 2.

### OR cleaning and disinfection standards

In 89% of the hospitals, plans for OR cleaning and disinfection after “septic” procedures were established in writing. In addition, 81% of the hospitals stated that some MDRB were explicitly named in the hygiene plan and that appropriate IPC measures were described for these. 62% differentiated between “aseptic” and “septic” intermediate OR cleaning and disinfection in their hygiene plans and explicitly described the IPC measures to be carried out in each case. 20% of the hospitals stated that no such distinction was made.

Likewise, 80% of respondents explicitly named bacteria with potentially special IPC requirements in their hygiene plans and described the corresponding IPC measures to be implemented.

### Main OR for endoprostheses

**Fig. 3:**
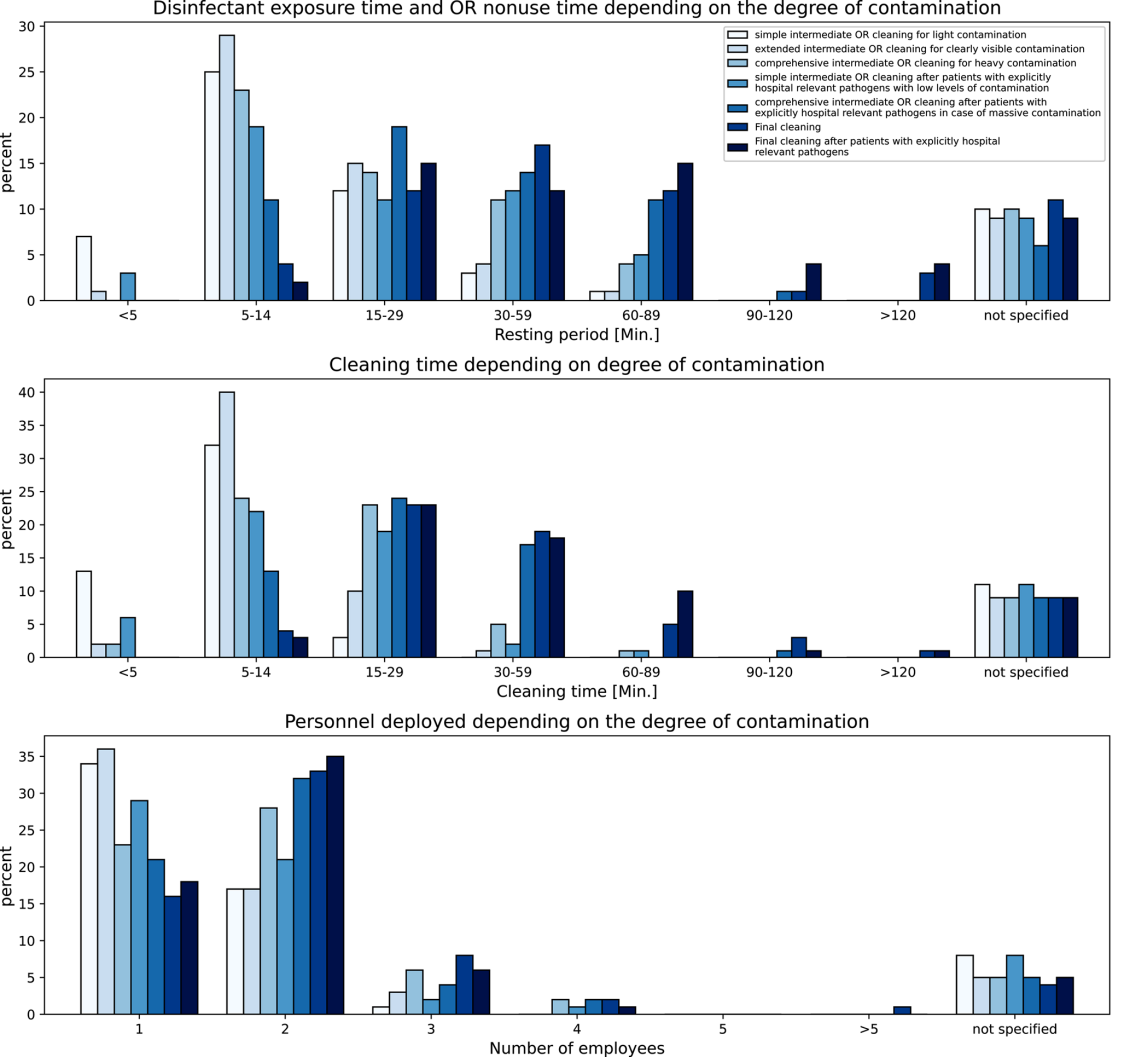
Duration of exposure time and, if applicable, OR nonuse time after reprocessing, as well as illustration of the deployment of cleaning and disinfection staff depending on the degree of contamination by potentially infectious materials, such as blood or excretions, of patients as assessed by the OR functional service. Note: The “final cleanings” are final cleanings at the end of the surgical program

In 62% of the participating hospitals, one or more ORs were available that were used exclusively for the implantation of endoprostheses. In 59% of the hospitals, “septic” patients also underwent surgery in these main ORs. Figure 3 shows the results for different process times and deployment of cleaning and disinfection staff depending on the degree of contamination. These degrees of contamination were established as an internal standard at our hospital then. Now they are part of the current guidelines issued by KRINKO in their 2022 “Hygiene Requirements for the Cleaning and Disinfection of Surfaces” [13].

### Postoperative isolation

**Fig. 4:**
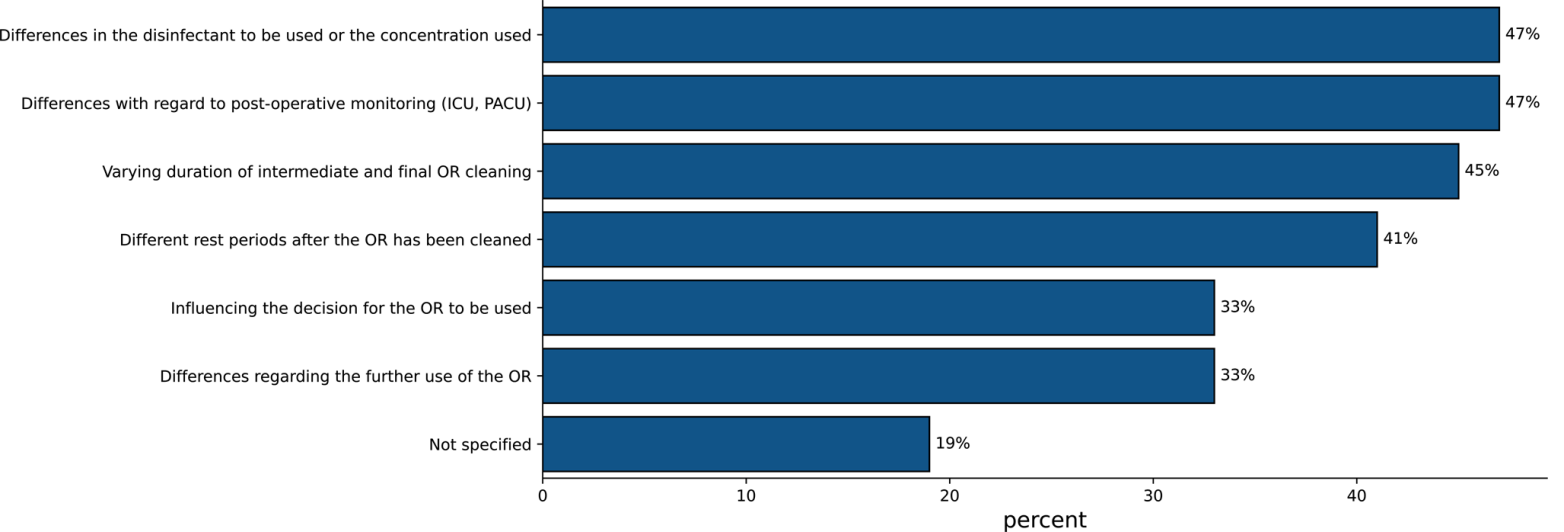
Postoperative monitoring of patients with explicitly hospital-relevant bacteria according to the type of pathogen and IPC requirements (multiple answers possible, *n* = 100)

In 56% of the participating hospitals, “septic” patients were isolated from other patients postoperatively (in the respective organizational unit post anesthesia care unit (PACU), intermediate care (IMC), and intensive care unit (ICU)), while 27% of the participants stated that this was handled “differently”. 8% of the respondents stated that there was no physical isolation, and 5% did not specify. Figure 4 shows the postoperative isolation results according to the pathogen spectrum.

Additional results of this survey are shown in Table 1 in the supplement.

## Discussion

This study provides concrete findings on the dissemination and application of current and historically evolved hygiene plans in the ORs of German hospitals. The results can serve as a basis for identifying specific needs for action, designing measures and determining the need for research to ultimately optimize patient care. The analysis showed that although the distribution of written hygiene plans is already high, it can and should be further improved. With regard to cost efficiency and limited OR capacities, the use of cost-intensive resources due to extended OR nonuse periods and postoperative follow-up care for patients in the OR for whom special IPC measures are required must be critically questioned.

Regarding the provision of written hygiene plans, most participants stated that these plans already exist. However, this was not the case for more than 20% of the participating hospitals and therefore reflects relevant potential for optimization. Although hygiene plans are clearly required by the German Infection Protection Act, an explicit requirement for the OR area is still missing [14, 15]. Irrespective of this, the provision of such a standard is clearly recommended, particularly in the OR, to prevent SSI and for quality assurance. An amendment to the corresponding Infection Protection Act should be discussed.

Almost 20% of the hospitals stated that hygiene plans were not the same for all departments. This is incomprehensible from an IPC perspective, as the aim of IPC measures in the OR should always be to create an environment with as few germs as possible. Therefore, these measures must be suitable for achieving this goal reliably, regardless of whether the cleaning is intermediate or final.

Our results showed that just under 1/3 of the hospitals surveyed had one or more ORs that were reserved exclusively for the treatment of “septic” patients. This provision is consistently demanded by the DGUV [16, 17]. However, the rather limited evidence available to date does not provide any indications of an advantage from such a provision [18, 19]. In 2019, a systematic literature review was conducted that revealed no significant improvements in terms of patient-related outcomes associated with physical separation [20]. According to the current Commission for hospital hygiene and infection prevention (KRINKO) recommendation for the prevention of SSI, there is also no evidence to date that separating ORs according to contamination class (“septic” OR) can reduce the risk of SSIs [4]. However, the provision and structural realization of such spatial separations are associated with considerable costs and can result in relevant process delays. Because there is no evidence of a positive influence on patient-related endpoints and because of the strained financial situation of German hospitals, such requirements should be critically scrutinized.

In most cases, the preoperative colonization status of a patient is not known, so it must always be assumed that colonization (or infection) could be present and must be acted upon. Consequently, standard IPC measures are of the utmost importance. Irrespective of the knowledge of the presence of particular bacteria, sufficient hygienic reprocessing of the OR is always necessary after every procedure to ensure hygienic and safe treatment of the subsequent patient.

Most respondents stated that they only operated on patients with explicitly hospital-relevant bacteria at the end of the OR schedule. This practice is probably intended to minimize possible contamination of other patients or the OR. Such a procedure suggests an insufficiency of IPC measures after interventions among these patients. However, this should not be the case if the relevant hygiene standards are applied correctly, regardless of patient order, and should rather lead to questioning the implementation of hygiene plans.

There is also an increased risk that patients can no longer be operated on at the end of the surgical program due to the advanced time of day and limited surgical resources. It is important to consider the impact of locally defined hygiene plans or traditional practices not required by IPC on treatment procedures and outcomes. Delays and therefore operations in the afternoon and evening hours can lead to an increased risk of morbidity or mortality [21–24]. Furthermore, this approach may not sufficiently consider the urgency of the procedure or the health condition of individual patients. Taken together, there is a risk of systematic discrimination against patients with explicitly hospital-relevant bacteria, which must be prevented. For these reasons, scheduling such procedures at the end of a regular surgical program must be viewed critically and should be avoided.

Approximately half of the participating hospitals defined the length of time the OR could not be used in accordance with the KRINKO guidelines until the floors and surfaces were dry [13]. Notably, around 1/3 stated that they waited at least 30 min after cleaning and disinfection before using the OR again. This unnecessary delay from an IPC point of view with a reduction in OR capacity, which is often already limited, can no longer be justified today with the economic burdens of German hospitals.

Almost all the participating hospitals stated that they had defined separate, written hygiene plans for OR cleaning and disinfection after “septic” operations and that they explicitly addressed some MDRO. However, since most patients, if they have no clinical signs of infection, do not have a differentiated pathogen status, and the cleaning and disinfection measures for MDRO do not primarily differ from those for antibiotic-sensitive bacteria, this differentiation between aseptic and septic intermediate OR cleaning and disinfection should be critically discussed. Finally, regular interim cleaning and disinfection of the OR should also cover the possible colonization of patients with explicitly hospital-relevant bacteria.

Cleaning must be distinguished from OR cleaning and disinfection after surgical procedures for patients with bacteria with special IPC requirements. For example, there are individual bacteria that are not or are not sufficiently removed with the usual disinfectants. Most hospitals had a corresponding standard as well. Conversely, 20% of hospitals did not have a standard for these bacteria and therefore still have potential for development.

The results of our survey showed that in 2/3 of the participating hospitals, one or more ORs were reserved specifically for the implantation of endoprostheses. Again, 2/3 of these hospitals also operated on “septic” patients in these ORs. Since there is no evidence to date of actual advantages for patient outcomes, the provision of special ORs for arthroplasty procedures does not make sense from an IPC perspective.

Regarding postoperative isolation measures, the results showed that most participating hospitals isolated “septic” patients from other patients postoperatively. However, why a small proportion of participants do not pursue a corresponding postoperative strategy remains unclear.

The location of postoperative care is particularly relevant because, depending on the pathogen, up to 1/3 of patients receive postoperative care in the OR, while almost ½ of those patients receive postoperative care in the intensive care or intermediate care unit. This means that relevant OR capacity and staff resources are used for the follow-up care of such patients, which does not lead to additional revenue, and at the same time, the staff are not available for the surgical care of other patients. In recent years, a pronounced shortage of nursing staff resources and thus bed capacity has developed in ORs and *high-dependency units.* Therefore, the postoperative use of these units for the isolation of such patients and the associated delayed care of other patients with an actual medical indication for postoperative intensive treatment is no longer justifiable, even from a safety standpoint.

According to the available results, only a small proportion of “septic” patients are monitored postoperatively in the PACU. Based on these findings, it is particularly important in the context of the targeted use of resources to further expand the PACU and to extend it both structurally and in terms of personnel so that the postoperative monitoring and isolation of such patients can be carried out there to a greater extent. When the PACU is not yet fully utilized (e.g., in the morning), it can also serve as a preoperative holding area. This allows patients to be sent to the OR at an early stage, thereby avoiding delays caused by intrahospital patient transport.

### Limitations

One limitation of the present study with regard to representativeness is the study design, since in the context of such surveys, it is sometimes the hospitals that are particularly well positioned in terms of the respective topic that respond, or in some cases, more “socially desirable” answers can be given [25].It is possible that the results presented may be more favorable due to this selective survey bias.

Another potential limitation is the choice of hospitals contacted. The hospitals selected for this study included those that also act as calculation hospitals and transmit their data to the InEK. This assumes a certain representativeness regarding German hospitals. However, it is not possible to definitively say whether the results regarding OR procedures are generalizable. In addition, smaller hospitals with fewer than 300 beds, as well as private and nonprofit hospitals, tend to be underrepresented in this collective [26].

It is important to note that national recommendations and guidelines have been significantly updated and refined since 2016. The KRINKO guidelines are very important for the German healthcare system and the implementation is also monitored by the local supervisory authorities.

Significant updates and specifications recommend to summarize important infection prevention precautions in bundles which have to be implemented, trained and supervised, always regarding the in-house regulations and specifications (“know your own hospital”) [4, 27]: It has been shown that protocol compliance and staff adherence to implemented requirements can be improved through training, i.e. regarding hand hygiene, perioperative antibiotic prophylaxis (PAP), behavior in the OR, surgical technique and others.

However, the changes to the guidelines listed as examples do not affect the topics of our survey. Therefore, this does not change the significance of our results.

Another potential limitation of our study comes from the fact that a definitive universally accepted definition for “septic” in the context of IPC is lacking. In this study, “septic” interventions refer to surgical procedures on patients colonized or infected with hospital-relevant pathogens, regardless of the infection site. The lack of a standardized definition of “septic” could lead to varying interpretations across clinical and research settings. By establishing a specific definition, we aim to enhance the clarity and consistency of our study’s focus on infection control in surgical environments. Our definition aims to ensure clarity in our investigation focused on infection control in surgical settings. The absence of a standardized definition underscores the need for further dialogue and potential standardization to improve infectious disease management and research consistency.

## Conclusions

Our study results showed that written hygiene plans are largely established in German hospitals. Nevertheless, there is some potential for improvement to further optimize the implementation of these plans. This includes the avoidance of unnecessarily extended nonuse periods in the OR after IPC measures and the postoperative care of patients with special IPC requirements in the OR and high-dependency units. At present, this approach often leads to a non-cost-covering use of resources.

In addition, our study indicates that non-evidence-based practices are still widespread. This emphasizes the need for further research and a focus on the implementation of evidence-based measures in the field of hospital hygiene.

Due to the national and international recommendations that have since been updated and the resulting revised guidelines and recommendations concerning infection prevention and infection control it would be useful to repeat the survey and evaluate not only the extent to which the participating hospitals have implemented the new guidelines, but also whether perception has changed in this regard due to the relevance for infection prevention and whether there is a higher response rate as in 2016. In this context, our existing data can serve as baseline data, providing a reference point against which new findings can be compared, thereby enhancing the understanding of changes over time and the impact of revised guidelines.

### Electronic supplementary material

Below is the link to the electronic supplementary material.

Supplementary Material 1

Supplementary Material 2

## Data Availability

https://docs.google.com/spreadsheets/d/e/2PACX-1vRi6JI9D09ODhnkqpX9UHdGzkoepJhKautJlN8nlaenjRP-kU-tRGHnWVQDxOzwCJfNe7ymAEVgwPtS/pubhtml.
