# Supplementary material for: Evaluation of the practice of reprocessing ORs in German hospitals from an infection prevention and control perspective
Source: Infection. 2024 Jun 3;52(4):1575–84. doi: 10.1007/s15010-024-02303-z (PMC11289043; doi:10.1007/s15010-024-02303-z)
Supplement: Supplementary file 1 — Supplementary Material 1 [file 15010_2024_2303_MOESM1_ESM.pdf]

Responses (n = 100)	
Hospital size (number of beds)	
250-499	34
500-1000	29
<250	24
>1000	12
Medical specialties working in your OR (multiple answers possible)	
General surgery	87
Trauma surgery/orthopedics	92
Urology	56
Neurosurgery	41
Thoracic surgery	34
Oral and maxillofacial surgery	36
Ophthalmology	35
Gynecology	71
Pediatric surgery	32
Plastic surgery	37
Cardiac surgery	20
Vascular surgery	57
Otorhinolaryngology	54
Dermatology	21
Involvement in procedures of the German Social Accident Insurance (DGUV) (multiple answers possible)	
Inpatient accident insurance doctor procedure	66

Injury type procedure	33
Major injury type procedure	25
None	9
Who is responsible for creating the hygiene protocols and guidelines in your hospital?	
Central department of hospital hygiene	37
Staff unit/ Organizational unit for hygiene including hospital hygiene	24
Infection prevention and control (IPC) specialists	2
Professional hygiene personnel	30
Department of microbiology	1
OR management	1
External consulting institute of hygiene	1
Other	1
Not defined	2
Who is responsible for implementing the hygiene protocols and guidelines in your hospital?	
Central department of hospital hygiene	20
Staff unit/ Organizational unit for hygiene including hospital hygiene	12
Infection prevention and control (IPC) specialists	5
Professional hygiene personnel	18
Department of microbiology	1
OR management	23
External consulting institute of hygiene	0
Other	15
Not defined	5
If yes, are responsibilities clearly defined in this hygiene instruction?	

Yes	73
No	8
Varies	7
When are these surgeries on patients with explicitly hospital relevant pathogens performed? (regarding endoprosthesis)	
At position 1 only if no subsequent endoprosthetic surgery is scheduled	1
During the day only if no subsequent endoprosthetic surgery is scheduled	4
No answer	6
Only at the end of the OR program	47
Regardless of position in the OR program	12
Is the maintenance of such a specifically isolated OR for patients with explicitly hospital relevant pathogens demanded by your Employer's liability insurance association?	
Yes	30
No	7
No answer	14
If you maintain an isolated OR for patients with explicitly hospital relevant pathogens, what types of surgeries are performed there?	
Exclusively surgeries on patients with multi-resistant pathogens (e.g., MRSA, VRE, MRGN)	1
Exclusively surgeries on patients with explicitly hospital relevant pathogens	4
Mixed	41
No answer	39
If mixed, what types of surgeries are performed in this OR?	
Aseptic	25
Surgeries on patients with explicitly hospital relevant pathogens	32
Surgeries on patients with multi-resistant pathogens (e.g., MRSA, VRE, MRGN)	35
No answer	36
Is this isolated OR for surgeries on patients with explicitly hospital relevant pathogens used by all surgical departments?	

Yes	41
No	14
No answer	33
Who informs the cleaning staff about an upcoming OR cleaning and disinfection after a surgical procedure?	
OR coordinator / OR manager	22
OR nurse	63
Anesthesia nurse	3
Other	1
Which professional group is responsible for determining the applicable cleaning and disinfection program in your ORs?	
OR coordinator / OR manager	25
OR nurse	53
Surgeon	1
Other	7
Not specified	3
Is the cleaning and disinfection of the Recovery room explicitly addressed in your hygiene instructions?	
Yes	61
No	17
Varies	5
No answer	6
Is the cleaning and disinfection of anesthesiological induction rooms or the central induction explicitly addressed in your hygiene instructions?	
Yes	65
No	17
Varies	4
No answer	3
Are special OR cleaning and disinfection s conducted before the surgical treatment of patients with latex allergy?	
Yes	11

No	60
No answer	18
At which organizational position in an OR are elective patients with a latex allergy operated on?	
Regardless of position, only the actual procedure is conducted latex-free	26
Only at first position	22
At any position, if latex-free procedures were used previously	7
Also at the second position, if latex-free procedures were used previously	5
No answer	29
What cleaning is carried out before a latex-free procedure if it has to be inserted as an emergency?	
Normal interim OR cleaning and disinfection	66
Interim cleaning after patients with explicitly hospital relevant pathogens	6
Final cleaning	6
Additional rest period for the OR (Please specify duration in minutes):	4
Is the Recovery room Part of the OR's Hygiene Area?	
Yes	59
No	27
No answer	3
If yes,...(is the Recovery room Part of the OR's Hygiene Area?)	
Are prepared beds from the regular ward used in the Recovery room?	30
Are unprepared beds from the regular ward used in the Recovery room?	24
Are internal OR beds used in the Recovery room?	6
Other procedures	5
Is the number of personnel needed for the respective cleaning and disinfection programs established?	
Yes	47
No	34
No answer	8

How long before the planned start of cleaning is the cleaning staff typically informed about the upcoming OR cleaning and disinfection (in minutes)?	
Median (25%-/75%-quartile)	15 (10/30)
Is the patient transferred from a regular ward bed to an OR table in the patient transfer area (and then transported to the OR)?	
Yes	85
No	1
Varies	1
Is the patient transferred from their hospital bed to the OR table inside the OR?	
Yes	4
No	74
Varies	1
Is the patient postoperatively transferred back to their hospital bed while still in the OR?	
Yes	7
No	74
Varies	4
If yes	
Back to their previous, prepared bed?	21
To a fresh bed from the central bed supply?	8
Back to their previous, unprepared bed from the regular ward?	4
To a fresh bed from the OR's bed pool?	0
