# Supplementary material for: Evaluation of the practice of reprocessing ORs in German hospitals from an infection prevention and control perspective
Source: Infection. 2024 Jun 3;52(4):1575–84. doi: 10.1007/s15010-024-02303-z (PMC11289043; doi:10.1007/s15010-024-02303-z)
Supplement: Supplementary file 2 — Supplementary Material 2 [file 15010_2024_2303_MOESM2_ESM.pdf]

Questionnaire

Questions and items used

General questions

1. **Distribution of ORs:**
    - How are the ORs distributed within your hospital?
        - Central ORs: ...
        - Decentralized ORs: ...
2. **Medical specialties working in your OR** (Multiple answers possible):
    - General surgery
    - Urology
    - Trauma surgery/orthopedics
    - Neurosurgery
    - Thoracic surgery
    - Oral and maxillofacial surgery
    - Ophthalmology
    - Gynecology
    - Pediatric surgery
    - Plastic surgery
    - Cardiac surgery
    - Vascular surgery
    - Otorhinolaryngology
    - Dermatology
3. **Size of your hospital:**
    - Less than 250 beds
    - 250-499 beds
    - 500-1000 beds
    - More than 1000 beds
4. **Level of care provided by your hospital:**
    - Primary and standard care
    - Special care
    - Specialized care
    - Maximum care
    - University hospital
5. **Involvement in procedures of the German Social Accident Insurance (DGUV)** (Multiple answers possible):
    - Inpatient accident insurance doctor procedure
    - Injury type procedure

- Major injury type procedure
- None

6. **Who is responsible for creating the hygiene plans in your hospital?**
    - Central department of hospital hygiene
    - Organisational unit for hygiene including hospital hygiene
    - Department of microbiology
    - OR management
    - Professional hygiene personnel
    - Infection prevention and control (IPC) specialists
    - External consulting institute of hygiene
    - External infection prevention and control (IPC) specialists
    - Other
    - Not defined
7. **Who is responsible for implementing the hygiene plans in your hospital?**
    - Central department of hospital hygiene
    - Organisational unit for hygiene including hospital hygiene
    - Department of microbiology
    - OR management
    - Professional hygiene personnel
    - Infection prevention and control (IPC) specialists
    - External consulting institute of hygiene
    - External infection prevention and control (IPC) specialists
    - Other
    - Not defined

Questions regarding IPC management in the OR

8. - Are there written hygiene plans for the OR at your hospital with detailed information on the type, extent, and duration of intermediate or final cleaning and the necessary staff?
        - Yes
        - No
        - Varies
        - No answer
9. - If yes, are responsibilities clearly defined in this hygiene plan?
        - Yes
        - No
        - Varies
        - No answer
10. - Are the hygiene plans identical for all medical specialties?
        - Yes
        - No
        - No answer
11. - Do you maintain one or more dedicated ORs exclusively for the implantation of endoprostheses?
        - Yes
        - No
        - No answer

12. • Are surgeries on patients with explicitly hospital relevant pathogens also performed in these dedicated ORs for the implantation of endoprostheses?
    - Yes
    - No
    - No answer

13. • When are these surgeries on patients with explicitly hospital relevant pathogens performed?
    - Regardless of position in the OR program
    - Only at the end of the OR program
    - At position 1 only if no subsequent endoprosthetic surgery is scheduled
    - During the day only if no subsequent endoprosthetic surgery is scheduled
    - No answer

14. • Do you differentiate ORs for surgeries on patients colonized or infected with explicitly hospital relevant pathogens regarding:
    - Choice of the OR to be used?
    - Disinfectants used and their concentration?
    - Duration of intermediate or final cleaning of the OR?
    - Downtime after cleaning and disinfection is completed?
    - Further use of the used OR?
    - Postoperative monitoring (Recovery room/Intensive care)?
    - No answer

15. • Are surgeries on patients with explicitly hospital relevant pathogens also performed outside of isolated 'septic' ORs?
    - Yes
    - No
    - No answer

16. • If patients with explicitly hospital relevant pathogens are operated on in aseptic ORs, then:
    - Regardless of position in the OR program
    - Only at the end of the OR program
    - At position 1 only if no subsequent surgery is scheduled
    - During the day only if no subsequent surgery is scheduled
    - No answer

17. • Do you have one or more ORs specifically reserved for surgeries on patients with explicitly hospital relevant pathogens?
    - Yes
    - No (please proceed to question 19)
    - No answer

18. • Is the maintenance of such a specifically isolated OR for patients with explicitly hospital relevant pathogens demanded by the DGUV in your case?
    - Yes
    - No
    - No answer

19. • If you maintain an isolated OR for patients with explicitly hospital relevant pathogens, what types of surgeries are performed there?
    - Exclusively surgeries on patients with explicitly hospital relevant pathogens
    - Exclusively surgeries on patients with MDRO (e.g., MRSA, VRE, MRGN)
    - Mixed
    - No answer

20. • If mixed, what types of surgeries are performed in this OR?
    - Aseptic

- Surgeries on patients with explicitly hospital relevant pathogens
- Surgeries on patients with MDRO (e.g., MRSA, VRE, MRGN)
- No answer

21. • Is this isolated OR for surgeries on patients with explicitly hospital relevant pathogens used by all surgical departments?
    - Yes
    - No
    - No answer

22. • Do you have written standards for OR cleaning and disinfection after surgeries on a patient with explicitly hospital relevant pathogens?
    - Yes
    - No
    - Varies
    - No answer

23. • Are there local standards for how long cleaned ORs cannot be used after a surgery on a patient with explicitly hospital relevant pathogens?
    - No
    - Until the floor and surfaces are dry
    - 10 minutes
    - 30 minutes
    - Longer than 30 minutes
    - No answer

24. • Where are patients with explicitly hospital relevant pathogens monitored postoperatively after the anesthesia ends? (Multiple answers possible)

	OR	PACU	IMC	ICU	No answer
General explicitly hospital-relevant pathogens					
MDRO (e.g., MRSA, MRGN, VRE)					
Colonizing/infecting bacteria with special IPC requirements					

25. • Are patients with explicitly hospital relevant pathogens postoperatively isolated from other patients in the respective unit (Recovery room, intermediate care unit, intensive care unit)?
    - Yes
    - No
    - Varies
    - No answer

Organizational Aspects

26. • Who informs the cleaning staff about an upcoming OR cleaning and disinfection after a surgical procedure?
    - OR nurse
    - Anesthesia nurse
    - Surgeon
    - Anesthetist
    - OR coordinator / OR manager
    - Other
    - Not specified
    - No answer

27. • Which professional group is responsible for determining the applicable cleaning and disinfection program in your ORs?
    - OR nurse
    - Anesthesia nurse
    - Surgeon
    - Anesthetist
    - OR coordinator / OR manager
    - Other
    - Not specified
    - No answer

28. • How long before the planned start of cleaning is the cleaning staff typically informed about the upcoming OR cleaning and disinfection (in minutes)?
    ...

29. • Is the number of personnel needed for the respective cleaning and disinfection programs established?
    - Yes
    - No
    - No answer

30. • Is the cleaning and disinfection of the Recovery room explicitly addressed in your hygiene plans?
    - Yes
    - No
    - Varies
    - No answer

31. • Is the cleaning and disinfection of anesthesiological induction rooms or the central induction explicitly addressed in your hygiene plans?
    - Yes
    - No
    - Varies
    - No answer

32. • Are special OR cleaning and disinfection s conducted before the surgical treatment of patients with latex allergy?
    - Yes
    - No
    - No answer

33. • At which organizational position in an OR are elective patients with a latex allergy operated on?
    - Only at the first position
    - Also at the second position, if latex-free procedures were used previously
    - At any position, if latex-free procedures were used previously
    - Regardless of position, only the actual procedure is conducted latex-free
    - No answer

34. • What cleaning is carried out before a latex-free procedure if it has to be inserted as an emergency?
    - Normal intermediate OR cleaning and disinfection
    - intermediate cleaning after patients with explicitly hospital relevant pathogens
    - Final cleaning
    - Additional rest period for the OR (Please specify duration in minutes):

35. A) Is the patient transferred from a regular ward bed to an OR table in the patient transfer area (and then transported to the OR)?
    - Yes
    - No
    - Varies
    - No answer

B) Is the patient transferred from their hospital bed to the OR table inside the OR?

- Yes
- No
- Varies
- No answer

C) Is the patient postoperatively transferred back to their hospital bed while still in the OR?

- Yes
- No
- Varies
- No answer

D) If yes to C):

- Back to their previous, unprepared bed from the regular ward?
- Back to their previous, prepared bed?
- To a fresh bed from the central bed supply?
- To a fresh bed from the OR's bed pool?

36. • Is the Recovery room Part of the OR's Hygiene Area?
    - Yes
    - No
    - No answer
  • If yes. . . :
    - Are unprepared beds from the regular ward used in the Recovery room?
    - Are prepared beds from the regular ward used in the Recovery room?
    - Are internal OR beds used in the Recovery room?
    - Other procedures

37. • Have you clearly differentiated in your hygiene plans between an aseptic intermediate OR cleaning and disinfection and disinfection and one after patients with explicitly hospital relevant pathogens, and described the respective hygiene measures to be carried out?
    - Yes
    - No
    - Varies
    - No answer

38. • Have you explicitly named some of the MDRO in the hygiene plans and described the corresponding hygiene measures to be carried out?
    - Yes
    - No
    - Varies
    - No answer

39. • Have you explicitly named pathogens with potentially special hygiene requirements in the hygiene plans and described the corresponding hygiene measures to be carried out?
    - Yes
    - No
    - Varies
    - No answer

40. • For aseptic conditions, which disinfectant(s) do you use for surface disinfection? (please select the concentration used, multiple responses possible)
    - Alcohols (≥70% / <70%)
    - Quaternary ammonium compounds (QUATs) (<1%; 1%, 2%, >2%)
    - Aldehydes/aldehyde releasers (0.5%; 1%; 2%; >2%)
    - Phenol (or derivatives) (<1.0%; 1.0%; 2.0%; >2.0%)
    - Chlorine-releasing compounds (0.5%; 0.75%; >1.0%)

- Oxidizing agents/peroxides (e.g., H2O2) (0.25%; 0.5%; 1.0%; >1.0%)

- For general pathogens with hospital relevance, which disinfectant(s) do you use for surface disinfection? (please select the concentration used, multiple responses possible)
  - Alcohols (≥70% / <70%)
  - Quaternary ammonium compounds (QUATs) (<1%; 1%, 2%, >2%)
  - Aldehydes/aldehyde releasers (0.5%; 1%; 2%; >2%)
  - Phenol (or derivatives) (<1.0%; 1.0%; 2.0%; >2.0%)
  - Chlorine-releasing compounds (0.5%; 0.75%; >1.0%)
  - Oxidizing agents/peroxides (e.g., H2O2) (0.25%; 0.5%; 1.0%; >1.0%)
- For MDRO such as MRSA, MRGN, VRE, which disinfectant(s) do you use for surface disinfection? (please select the concentration used, multiple responses possible)
  - Alcohols (≥70% / <70%)
  - Quaternary ammonium compounds (QUATs) (<1%; 1%, 2%, >2%)
  - Aldehydes/aldehyde releasers (0.5%; 1%; 2%; >2%)
  - Phenol (or derivatives) (<1.0%; 1.0%; 2.0%; >2.0%)
  - Chlorine-releasing compounds (0.5%; 0.75%; >1.0%)
  - Oxidizing agents/peroxides (e.g., H2O2) (0.25%; 0.5%; 1.0%; >1.0%)
- For pathogens with special hygiene requirements such as spore formers, noroviruses, etc., which disinfectant(s) do you use for surface disinfection? (please select the concentration used, multiple responses possible)
  - Alcohols (≥70% / <70%)
  - Quaternary ammonium compounds (QUATs) (<1%; 1%, 2%, >2%)
  - Aldehydes/aldehyde releasers (0.5%; 1%; 2%; >2%)
  - Phenol (or derivatives) (<1.0%; 1.0%; 2.0%; >2.0%)
  - Chlorine-releasing compounds (0.5%; 0.75%; >1.0%)
  - Oxidizing agents/peroxides (e.g., H2O2) (0.25%; 0.5%; 1.0%; >1.0%)

1 Hygiene measures

Definitions:

A. **Simple intermediate OR cleaning and disinfection:**
Cleaning and disinfection measures include visibly dirty and contaminated objects and contact surfaces. Additionally:
- Exchange OR table
- Dispose of laundry and waste
- Clean washing room sink

B. **Extended intermediate OR cleaning and disinfection:**
In addition to simple intermediate OR cleaning and disinfection, the following are cleaned:
- Floor as needed
- Walls as needed with targeted cleaning and disinfection
- OR lamps including handles
- All positioning aids
- Instrumentation/side tables
- X-ray aprons
- Medical-technical devices, as used (e.g., suction devices)

C. **Comprehensive intermediate OR cleaning and disinfection:**
In addition to extended intermediate OR cleaning and disinfection, the following are cleaned:
- Floor within the movement radius
- Washing room and disposal
- All positioning aids
- X-ray aprons, X-ray viewers, X-ray equipment
- Medical-technical devices including cables/control panels
- Cabinets outside and handles
- Rollboard and OR chair

D. **Simple intermediate OR cleaning and disinfection after patients with explicitly hospital relevant pathogens:**
Cleaning and disinfection measures include the immediate OR area and visibly dirty and contaminated objects and surfaces. Additionally:
- OR table remains in the room, exchanged after cleaning and disinfection on-site
- Positioning aids/cushions
- Clean washing room sink
- Dispose of laundry and waste

E. **Comprehensive intermediate OR cleaning and disinfection after patients with explicitly hospital relevant pathogens:**
In addition to simple intermediate OR cleaning and disinfection, the following are cleaned:
- OR lamps including handles
- Floor within the movement radius, washing room and disposal
- All positioning aids
- Instrumentation tables
- X-ray aprons, X-ray viewers, X-ray equipment
- Medical-technical devices including cables/control panels (e.g., suction devices)
- Cabinets outside and handles
- Rollboard and OR chair

41. How many cleaning staff members are allocated for each of the above categories?

   A. Number of staff for simple intermediate OR cleaning and disinfection (minor contamination):
   B. Number of staff for extended intermediate OR cleaning and disinfection (visible contamination):
   C. Number of staff for comprehensive intermediate OR cleaning and disinfection (massive contamination):
   D. Number of staff for simple OR cleaning and disinfection after hospital relevant pathogens (minor contamination):
   E. Number of staff for comprehensive OR cleaning and disinfection after hospital relevant pathogens (massive contamination):
   F. Number of staff for final cleaning:
   G. Number of staff for final cleaning after hospital relevant pathogens:

42. How much time is allocated for each type of OR cleaning and disinfection and disinfection ?

   A. Time allocated for simple intermediate OR cleaning and disinfection (minor contamination):
   B. Time allocated for extended intermediate OR cleaning and disinfection (visible contamination):
   C. Time allocated for comprehensive intermediate OR cleaning and disinfection (massive contamination):
   D. Time allocated for simple OR cleaning and disinfection after patients with explicitly hospital relevant pathogens (minor contamination):
   E. Time allocated for comprehensive OR cleaning and disinfection after patients with explicitly hospital relevant pathogens (massive contamination):
   F. Time allocated for for final cleaning:
   G. Time allocated for final cleaning after hospital relevant pathogens:

43. How long is the time interval during which an OR cannot be used for patient care due to cleaning and disinfection measures for each category?

   A. Time interval for OR unavailability after simple intermediate OR cleaning and disinfection (minor contamination):
   B. Time interval for OR unavailability after extended intermediate OR cleaning and disinfection (visible contamination):
   C. Time interval for OR unavailability after comprehensive intermediate OR cleaning and disinfection (massive contamination):
   D. Time interval for OR unavailability after simple OR cleaning and disinfection after hospital relevant pathogens (minor contamination):
   E. Time interval for OR unavailability after comprehensive OR cleaning and disinfection after hospital relevant pathogens (massive contamination):
